# Identifying similarities at metabolic pathways with a strategy of Enzymatic Step Sequences

**DOI:** 10.1016/j.mex.2023.102118

**Published:** 2023-03-11

**Authors:** Augusto Cesar Poot-Hernandez, Katya Rodriguez-Vazquez, Ernesto Perez-Rueda

**Affiliations:** a Unidad de Bioinformática y Manejo de la Información. Instituto de Fisiología Celular. Universidad Nacional Autónoma de México, Ciudad Universitaria, México, Mexico; b Departamento de Ingeniería de Sistemas Computacionales y Automatización, Instituto de Investigaciones en Matemáticas Aplicadas y en Sistemas, Universidad Nacional Autónoma de México, Ciudad Universitaria, México, Mexico; c Instituto de Investigaciones en Matemáticas Aplicadas y en Sistemas, Universidad Nacional Autónoma de México, Unidad Académica del Estado de Yucatán. Mérida, Yucatán. Mexico

**Keywords:** Enzymatic Step Sequences (ESS) comparative program, Enzyme commission number, Metabolic pathways, Enzymatic step sequences, KEGG, Comparative genomics

## Abstract

An easy and fast strategy to compare functionally the metabolic maps is described. The KEGG metabolic maps are transformed into linear Enzymatic Step Sequences (ESS) using the Breadth First Search (BFS) algorithm. To do this, the KGML files are retrieved, and directed graph representations are created; where the nodes represent enzymes or enzymatic complexes, and the edges represent a compound, that is the 'product' from one reaction and a 'substrate' for the next. Then, a set of initialization nodes are selected, and used as the root for the construction of the BFS tree. This tree is used as a guide to the construction of the ESS. From each leaf (terminal node), the path is traced backwards until it reaches the root metabolic map and with two or fewer neighbors in the graph. In a second step, the ESS are compared with a Dynamic Programing algorithm, considering an “ad hoc” substitution matrix, and minimizing the global score. The dissimilarity values between two EC numbers ranged from 0 to 1, where 0 indicates similar EC numbers, and 1 indicates different EC numbers. Finally, the alignment is evaluated by using the normalized entropy-based function, considering a threshold of ≤ 0.27 as significant.

• The KEGG metabolic maps are transformed into linear Enzymatic Step Sequences (ESS) using the Breadth First Search (BFS) algorithm.
• Nodes represent enzymes or enzymatic complexes, and the edges represent a compound, that is 'product' from one reaction and a 'substrate' for the next.
• The ESS are compared with a Dynamic Programing algorithm, considering an “ad hoc” substitution matrix, and minimizing the global score.

## Graphical abstract

**Figure:**
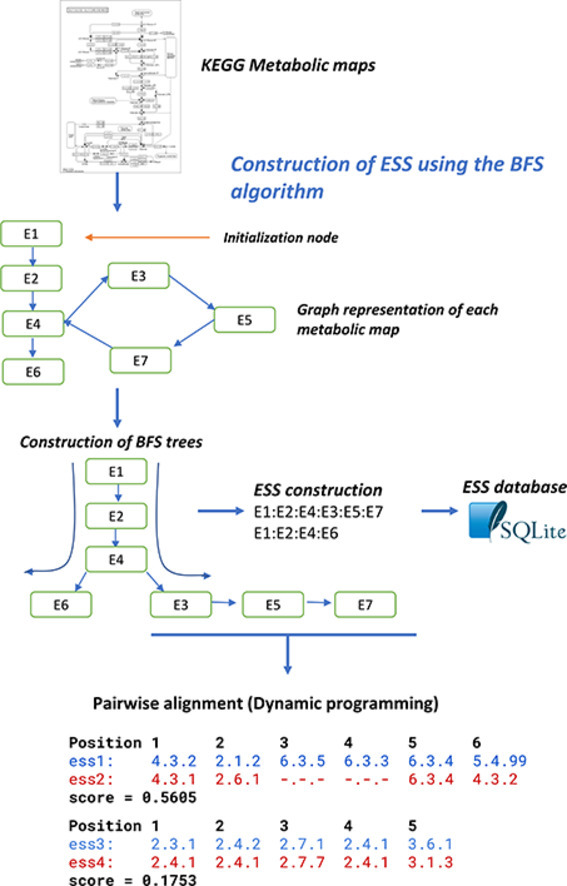

Specifications table

**Table:** 

Subject Area:	Biochemistry, Genetics and Molecular Biology
More specific subject area:	Metabolism, Microbiology, comparative genomics
Method name:	Enzymatic Step Sequences (ESS) comparative program
Name and reference of the original method:	Poot-Hernandez, A.C., Rodriguez-Vazquez, K. and Perez-Rueda, E. [3] The alignment of enzymatic steps reveals similar metabolic pathways and probable recruitment events in *Gammaproteobacteria. BMC Genomics***16**, 957.
Resource availability:	https://github.com/acpooth/essdb

## Method

Here, we present a method to functionally compare metabolic pathways obtained from the KEGG database. At first, the Kyoto Encyclopedia of Genes and Genomes *(KEGG) metabolic maps are converted into graphs where the nodes are enzymes and edges are product-substrate relationships. Then, the breadth-first search algorithm is used to extract linear Enzymatic Step Sequences (ESS) from the metabolic map graphs, and store them in a database. These ESS represent consecutive proteins or complexes of proteins with enzymatic roles, functionally linked to each other, where their catalytic activities are represented by the Enzyme Commission numbers. In a second step, pairwise comparisons of these ESS are achieved by using the Needleman and Wunsch (dynamic programming) algorithm, to identify the most significant pairs of (functional) sequences. This article presents an update of the method published by Poot-Hernandez et al. [3] in the form of two GitHub repositories that facilitate their use by other researchers.

## Construction of enzymatic step sequences (ESS)

**Fig. 1:**
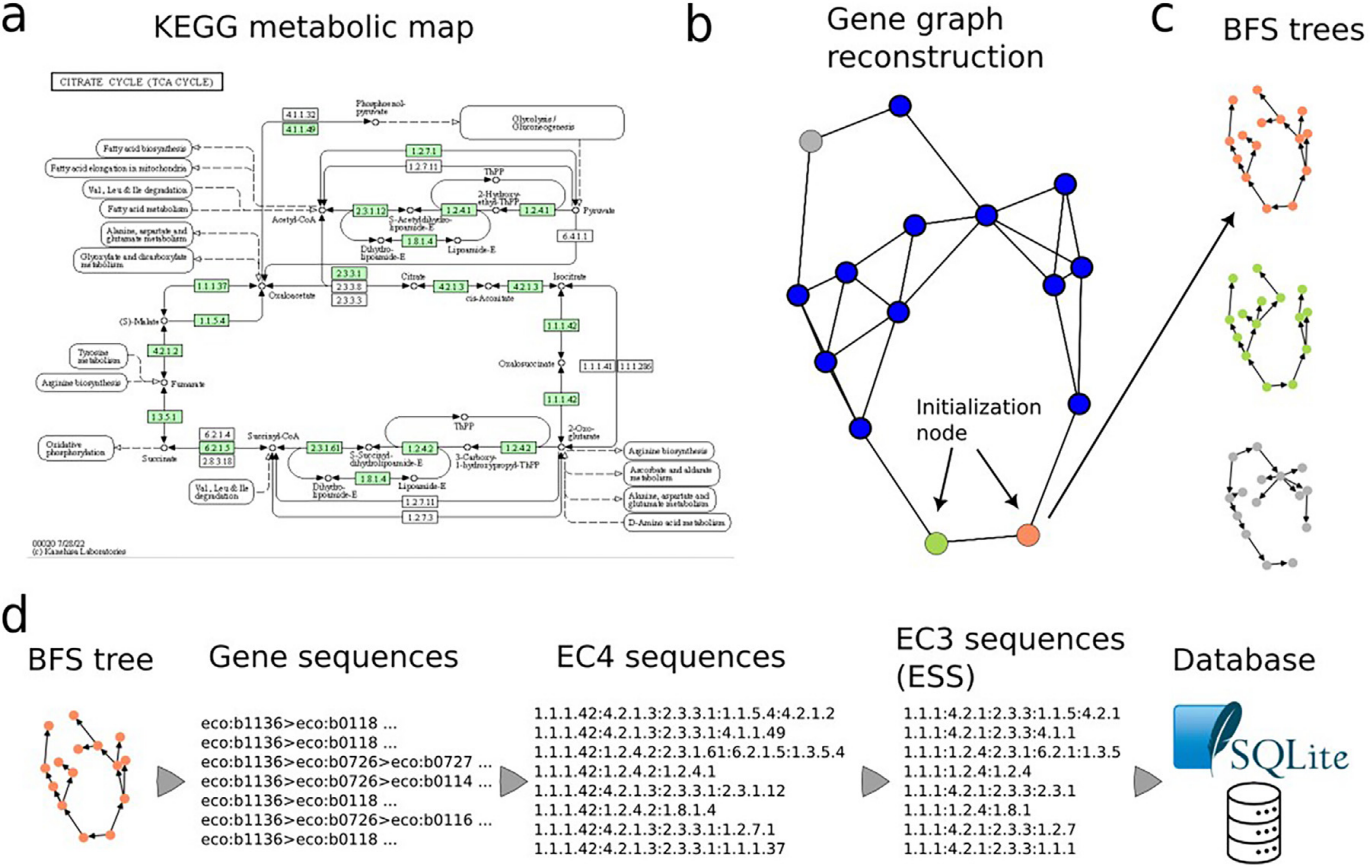
Workflow of the construction of ESS from KEGG metabolic maps. See text for details.

The metabolic maps stored in the KEGG database are used to create the database of ESSs. The first step is to download the KGML files that describe the metabolic maps (pathways, Fig. 1a). This method only works with strict metabolic maps, *i.e.,* those maps that describe consecutive enzymatic steps with a product/substrate relationship. These maps are easily recognized because the KEGG map code has numbers below “01,000″. For example, the map “00,010″ represents the Glycolysis/Gluconeogenesis pathway, whereas the map “00,230″ represents the Purine metabolism pathway. In these maps, an enzymatic reaction may represent a single enzyme or an enzymatic complex, and a single reaction may be represented by more than one gene product, such as isoenzymes.

To construct the ESS for these metabolic maps the Breadth First Search (BFS) algorithm is used. To do this, each metabolic map is converted into a graph in which the nodes represent the enzymatic reactions and the edges represent the product/substrate relationships between them (Fig. 1b). This means that two enzymes are connected if the product of one of them is the substrate of the other. In addition, the construction of the graph takes into account the reversibility of the reactions. The method can construct, as an option, directed graphs as in the graphical abstract or undirected graphs as in the Fig. 1b.

To use the BFS algorithm it is necessary to select a node that will be used as BFS tree root. We called these nodes “initialization nodes” (Fig. 1b). The initialization nodes for each metabolic map were selected considering the following criteria: (1) nodes whose substrates are not produced by any other reaction in the map; and (2) nodes connecting with other metabolic maps, and with a connectivity less than 3, that limit the number of ESS produced by a single metabolic map. Then, each initialization node is used to create a BFS tree (Fig. 1c). If the graph is directed, the BFS algorithm has the additional restriction of directionality to construct the tree, *i.e.,* it only follows the nodes in forward direction. For this reason, the directed graph option tends to create less ESS. The ESS are produced from each tree tracking the path from the leaves to the root node (Fig. 1d). The sequences of gene identifiers are translated into a sequence of EC numbers at four and three levels of EC classification. Each tree will produce as many sequences as leaves contain, and each metabolic map will produce as many BFS trees as initialization nodes contain. Therefore, metabolic maps with more enzymes tend to produce more ESSs. Because an ESS may be the same in different species or maps, *i.e.,* the ESS exhibits redundancy, a non-redundant dataset (nrESS) is constructed by filtering identical ESS and leaving only one representative.

The nrESSs are organized into a relational database, where each EC number contained in a sequence is related to its corresponding gene(s), organism species (Bacteria, Archaea or Eukarya), and metabolic map (KEGG). In addition, each nrESS is linked to the original ESS. Finally, all the ESSs alignments can be conducted using the nrESS dataset and referring to the original data when necessary. See Fig. 1.

All the scripts to create ESSs and the corresponding SQLIte database are public in a GitHub repository (https://github.com/acpooth/essdb) where the structure of the database is also described. The construction of the graphs and the BFS trees is conducted with Python scripts using the NetworkX module (Hagberg AA, et al. [1]).

## Comparison of nrESS by pairwise alignments

To compare and identify similarities between nrESS a pairwise alignment algorithm based on the Dynamic Programing Needleman and Wunsch (NW) algorithm has been implemented. This algorithm works in a similar way as the classic tools to align nucleotide or amino acid sequences with the difference that it evaluates the similarities of enzymatic steps using an entropy-based scoring function. This function was previously created by our group as part of the objective function of a genetic algorithm to build multiple ESS alignments [2] and adapted to the pairwise alignment of ESS using NW algorithm [3].

The function measures the homogeneity of a column in an alignment using the Shannon entropy equation. In this regard, each EC number is composed of 3 “columns”, each of which corresponds to the first 3 levels of the EC classification. As the function measures entropy, high entropy values mean different symbols in a column, whereas low entropy values mean similar symbols. In other words, as the function values increase, the similarity of EC numbers decreases, and *vice versa*. Thus, this function acts as a dissimilarity measure and it is calculated as follows.

## Dissimilarity of EC numbers

For the case of the multiple ESS alignments [2], the dissimilarity of two EC numbers, ECS(EC_1_*,*EC_2_), considering the first three classification levels (*l_1_.l_2_.l_3_*) is evaluated according the following equation:

$$ECS\left(EC1,EC2\right)=\frac{w_{1}H_{1}+w_{2}H_{2}+w_{3}H_{3}}{w_{1}+w_{2}+w_{3}}$$

Where *w_1_, w_2_, w_3_* correspond to the weight factors for each classification level, and *H_1_, H_2_, H_3_* to the entropy measured for each aligned level. The weight factors used were 15, 10 and 1 for the first, second and third classification levels respectively. These factors were selected based on the qualitative observation of the alignments generated. As mentioned above, the entropy for each classification level, *l*, was calculated according the following equation based in information theory [4]:

$$H_{1}=\sum_{i=1}^{s}p_{i}\log_{2}\left(p_{i}\right)$$

Where *s* is the number of symbols in the aligned level *l* and *p_i_* is the probability to find said symbol. The probability pi is calculated by column (EC level), dividing the number of occurrences of a symbol by the number of sequences. As mentioned before, this function was created for the evaluation of multiple ESS alignments, where there can be many symbols (EC numbers) in a column of an alignment. However, in the case of a pairwise alignment, there can be a maximum of 2 symbols and a minimum of 1 symbol per column (EC level). For this reason, we can simplify the calculation of the EC dissimilarity as follows: If the classifications of both EC numbers in *l* are equal, then *H_l_* = 0, if they are different then *H_l_ = 1*. Therefore, when the entropy value for two EC numbers is high, the dissimilarity is high. In contrast, when the entropy value is low, the dissimilarity is small.

For the special case of pairwise ESS alignment, the dynamic programming NW algorithm can be much faster than the genetic algorithm previously used [2]. Moreover, it requires a scoring matrix in order to evaluate the construction of the alignment, typically called a substitution matrix. This matrix contains the precomputation of the dissimilarity scoring of all the EC numbers. In this regard, the simplified method mentioned above is used to create an EC number dissimilarity (scoring) matrix, *S*. The matrix represents the pairwise comparison of 319 different 3 levels of the EC numbers stored in the KEGG database. The number 9.9.9 was added to describe enzymes with no EC assigned and it is similar only to itself.

Another advantage of the scoring matrix is that it can take into account the hierarchy of the EC numbers; for instance, giving a value of 1 to all the EC pairs that are different in the first level of classification regardless of whether the second or third numbers are identical. This also applies to the second classification level. For this reason, the score of the matrix S is different from the value obtained with theECS(EC1,EC2) equation. In this regard, the first is suitable for the pairwise alignment whereas the second is useful for the multiple ESS alignment. Some examples of EC dissimilarity in the scoring matrix *S* are: S_1.2.7_*_, 1.2.7_* = 0; S_1.2.7_*_, 3.2.2_* = 1; S_2.3.1_*_, 2.4.1_* = 0.423; S_4.3.1_*_, 4.3.2_* = 0.0384; *i.e.,* low entropy values (close to cero) suggest ESS similarity, whereas values close to 1, suggest different ESS*.*

## NW dynamic programming algorithm

To perform the NW algorithm of the ESS_1_ and ESS_2_ given a scoring matrix *S,* a DP matrix, *M*, with shape *n* x *m* is constructed. Where *n* is the length of ESS_1_ and *m* is the length of *ESS_2_*.The matrix *M* is filled according to the following rule. For each cell *M_i,j_*:

$$M_{i,j}=\min\left\{ \begin{array}{c} M_{i-1,j-1}+S_{EC_{i}EC_{j}}\\ M_{i,j-1}+gap\\ M_{i-1,j}+gap \end{array}\right.$$

Where *S_ECiECj_* is the value of dissimilarity assigned in the matrix *S* between the EC numbers *EC_i_* and *EC_j_*. The *gap* penalization value is set to 1, equal to the maximum dissimilarity between two EC numbers. In this way, the best values add up in the diagonal of the matrix *M* that represents the optimum alignment. Therefore, the best score of the matrix is traced back to build the alignment, adding gaps in the corresponding and optimal positions. The gaps are represented with the string “-.-.-” in the ESS alignment.

## Alignment scoring function

After the alignment is performed, it is evaluated using a normalized scoring function that allows to take into account the gaps and to restrict the score value in the range of 0 to 1. The score is calculated as follow:

score=0.95(H)+0.05(GP)

Where *H* is the mean homogeneity (entropy) of the aligned ESS and *GP* is the penalization for the gaps.

The homogeneity is measured by the equation:

$$H=\frac{\sum_{i=1}^{n}S_{EC_{i1}EC_{i2}}}{n}$$

Where *n* is the length of the alignment, and *S_ECiECj_* is the score of dissimilarity in the matrix *S* of the two EC numbers aligned in the position *i*. When an EC number is aligned with a gap (-.-.-), it adds up to the homogeneity as maximum dissimilarity (1). The gap penalization is measured by the equation:

$$GP=\frac{\sum_{i-1}^{ns}GB_{i}\;/\;TG_{i}}{ns}$$

Where *GB_i_* is the number of gap blocks in the ESS *i, TG_i_* is the total number of gap characters in the ESS *i* (the gap character is “-.-.-“) and *ns* is the number of ESS (2 for a pairwise alignment). This equation evaluates the mean concentration of the gaps. The numerator becomes smaller as the number of gap blocks increases, thus decreasing the gap penalization. The gaps at the beginning and the end of the ESSs are not included in the count of gaps for the gap penalization, but they are included in the homogeneity measure.

## Statistically significant of the ESS alignments

The statistical significance of the nrESS alignments, is determined by comparing the alignment scores of the real database against the scores from 10 different random databases. These random sequences were constructed by shuffling the EC number content of the entire database, maintaining the nrESS length and EC composition of the original ESS. Each random database was submitted to the same all-versus-all. alignment approach used for the real data, and the distribution of alignment scores considered the mean ± SD. The threshold considered statistically significant corresponded to a score of ≤ 0.27, *i.e.*, that point with higher dispersion of the real data relative to the mean random databases scores and where the loss of nrESS due to extreme dissimilarity was less than 1%. This score can be used as a “rule of thumb” and a good starting point to search for similarities in ESS.

## Examples of use

In the following, two examples of the command line tool are shown, where the ESS of the low part of the Glycolysis and the Inosine Monophosphate (IMP) pathways were compared and their similarities displayed.

## Documentation

***usage: alignESS.py pair [-h] [-l] ess1 ess2*** positional arguments:

ess1 ESS (3 levels EC numbers. Colon separated.
  (2.7.1:5.3.1:5.3.1: 2.7.1:4.1.2:1.2.1)
ess2 ESS (3 levels EC numbers). Colon separated.
  (5.3.1:5.3.1: sss4.2.1)

optional arguments:

-h, –help show this help message and exit
-l, –localize the alignment is trimmed to the coverage of the shortest ESS and the score is then calculated to the trimmed alignment. This method allows to find 'local-like' alignments between ESS of different sizes.

***$input***

alignESS.py pair 2.7.1:5.3.1:5.3.1:2.7.1:4.1.2:1.2.1 5.3.1:5.3.1:4.2.1

***$output***

ess1: 2.7.1:5.3.1:5.3.1:2.7.1:4.1.2:1.2.1

ess2: -.-.-:5.3.1:5.3.1:-.-.-:4.2.1:-.-.- score = 0.56698

The second example shows a comparison in an all *vs* all fashion in the complete ESS database, by using the following command:

## Documentation

***usage: alignESS.py dbalign [-h] [-db2 ESSDB2] [-o OUTFILE] [-t THRESHOLD] [-nproc NPROC] [-l] [-align] essdb1*** positional arguments:

essdb1 ESSs database 1. Sqlite3 file with nrseqs table or text file with one
  ESS in each line. If the essdb2 argument is not specified, the program performs the all-vs-all alignment in essdb1. This argument can also be a single ESS, in this case the -db2 argument is necessary.

optional arguments:

-h, –help show this help message and exit
-db2 ESSDB2, –essdb2 ESSDB2
  ESSs database 2. Sqlite3 file with nrseqs table or text file with one ESS in each line
-o OUTFILE, –outfile OUTFILE
  Outfile name to report scores. By default, the file only contains the id of the ESSs and the score of the alignment.
  If argument '-align' is set, then the file contains the aligned ESSs
-t THRESHOLD, –threshold THRESHOLD
  Threshold score to filter results in the range 0–1 [0.27]. If the threshold is high (>0.6) and the databases are large,
  results may saturate the
  RAM memory, beware!
-nproc NPROC Number of processes to execute analysis. It can be created more processes than cores in the processor, so the speedup
  of the analysis depends on the number of cores available
-l, –localize the alignment is trimmed to the coverage of the shortest ESS and the score is then calculated to the trimmed
  alignment. This method allows to find 'local-like' alignments between ESS of different size
-align If set, the outfile contains the alignment of each ESS pair below the threshold. Beware, if the databases are large
  and the threshold high, the file may be huge or the RAM memory will collapse.

***$input***

***alignESS.py dbalign seqs.db***

***$output***

Random sample of the output stored in file output.txt. By default, the database alignment option returns only the scores with values less than 0.27 (see below); *i.e.*, considered as significant:

**Table:** 

ess1	ess2	score
41,386	42,207	0.18066
109,507	111,623	0.24999
42,112	72,085	0.24179
116,852	117,122	0.16255
54,091	66,266	0.20096

## Declaration of Competing Interest

The authors declare that they have no known competing financial interests or personal relationships that could have appeared to influence the work reported in this paper.

## Data Availability

Data will be made available on request.
